# High Precision of Spike Timing across Olfactory Receptor Neurons Allows Rapid Odor Coding in *Drosophila*

**DOI:** 10.1016/j.isci.2018.05.009

**Published:** 2018-05-17

**Authors:** Alexander Egea-Weiss, Alpha Renner, Christoph J. Kleineidam, Paul Szyszka

**Affiliations:** 1 University of Konstanz, Department of Biology, Neurobiology, Konstanz 78457, Germany; 2 Institute of Neuroinformatics, University of Zurich and ETH Zurich, Zürich 8057, Switzerland

**Keywords:** Cellular Physiology, Neuroscience, Sensory Neuroscience

## Abstract

In recent years, it has become evident that olfaction is a fast sense, and millisecond short differences in stimulus onsets are used by animals to analyze their olfactory environment. In contrast, olfactory receptor neurons are thought to be relatively slow and temporally imprecise. These observations have led to a conundrum: how, then, can an animal resolve fast stimulus dynamics and smell with high temporal acuity? Using parallel recordings from olfactory receptor neurons in *Drosophila*, we found hitherto unknown fast and temporally precise odorant-evoked spike responses, with first spike latencies (relative to odorant arrival) down to 3 ms and with a SD below 1 ms. These data provide new upper bounds for the speed of olfactory processing and suggest that the insect olfactory system could use the precise spike timing for olfactory coding and computation, which can explain insects' rapid processing of temporal stimuli when encountering turbulent odor plumes.

## Highlights

• Olfactory receptor neuron responses are fast and temporally precise
• Odor-evoked spikes can occur 3 ms after odorant arrival and jitter less than 1 ms
• First-spike timing varies over a wider concentration range than spike rate
• Neural network model demonstrates the plausibility of a spike-timing code for odors

## Graphical Abstract

**Figure:**
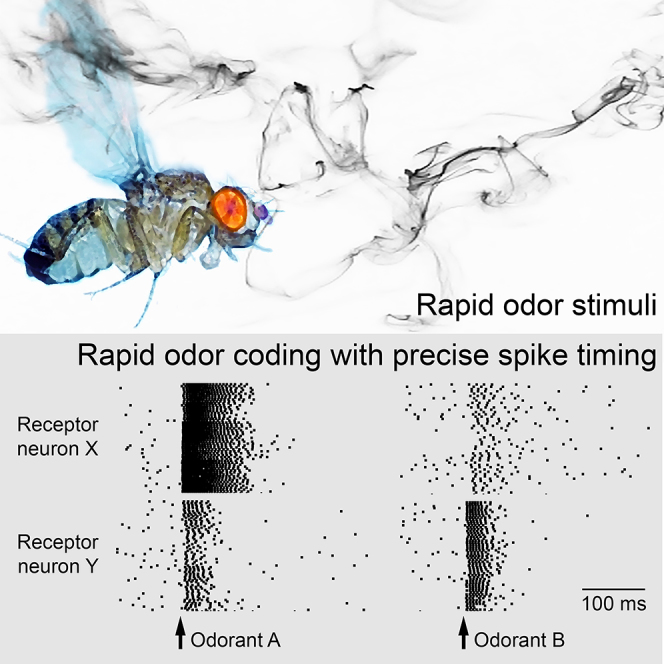

## Introduction

Olfaction is a highly dynamic process, as wind and self-generated movement expose olfactory organs to rapid changes in odorant concentrations (Celani et al., 2014, Farrell et al., 2002, Huston et al., 2015, Murlis, 1992, van Breugel and Dickinson, 2014, Vickers, 2000). Flying insects are particularly well adapted for rapidly detecting and tracking odorants in turbulent environments. *Drosophila*, for example, can react within 70 ms after the response onset of olfactory receptor neurons (Gaudry et al., 2013), and moths and honey bees use millisecond short differences in odorant arrival for odor source separation (Baker et al., 1998, Szyszka et al., 2012). These fast smelling capabilities imply a rapid neural coding mechanism for odors. The speed at which sensory systems can encode stimuli depends on the temporal precision of stimulus-evoked spikes: Higher temporal precision allows faster stimulus encoding, because postsynaptic neurons require shorter integration times to separate stimulus-evoked spikes from spontaneous spikes (Jeanne and Wilson, 2015, Thorpe et al., 2001). Accordingly, many sensory systems use millisecond or even sub-millisecond precise spike timing across sensory neurons to rapidly encode stimulus features (e.g., visual patterns in salamanders [Gollisch and Meister, 2008], direction of sound in barn owls [Carr and Konishi, 1990], and touch location in leeches [Thomson and Kristan, 2006]).

Odorant identity is encoded in the differences of spike rates and spike latencies across olfactory receptor neuron types (insects [de Bruyne et al., 1999, Martelli et al., 2013, Schneider et al., 1964]; vertebrates [Duchamp-Viret et al., 1999, Getchell and Shepherd, 1978]). However, the speed and the temporal precision of stimulus-evoked spikes in olfactory receptor neurons have not yet been accurately determined. Using parallel recordings from olfactory receptor neurons in *Drosophila*, we found fast and temporally precise odorant-evoked spike responses: The first odorant-evoked spike occurred with a short latency down to 3 ms and with a trial-to-trial and neuron-to-neuron SD (jitter) below 1 ms. Using a simple neural network model, we demonstrate the plausibility of a rank order code for odorant identity based on relative spike latencies across different receptor neuron types.

## Results and Discussion

**Figure 1:**
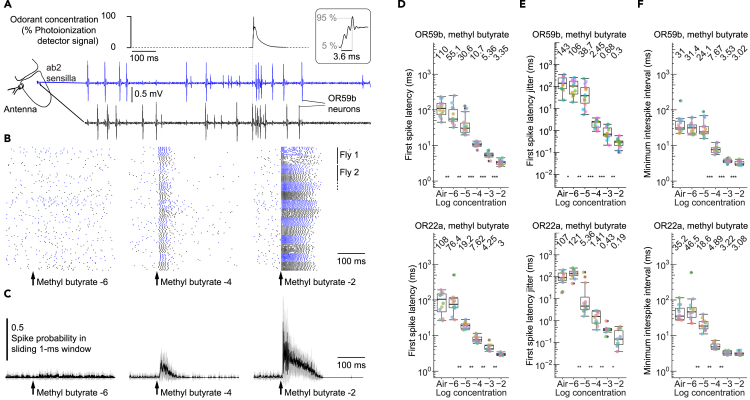
Timing of First Spikes in Olfactory Receptor Neurons Varies Over a Wider Concentration Range than Spike Rates (A) Simultaneous, extracellular recordings from two OR59b neurons in two different ab2 sensilla during a pulse of methyl butyrate at a concentration of 10^−4^. Top: Odorant stimulus measured with a photoionization detector. Timescale bar refers to both panels. Inset: Odorant onset (3.6 ms rise time, mean ± SD over ten stimulus repetitions). (B) Raster plot of spikes in seven simultaneously recorded pairs of OR59b neurons during stimulation with methyl butyrate at different concentrations (10^−6^, 10^−4^, 10^−2^). (C) Same data as in (B) presented as spike probability in a sliding 1 ms window (mean ± SD over 14 OR59b neurons). (D–F) First spike latencies (D), trial-to-trial jitter of first spike latencies (E), and minimum interspike intervals (1/maximum spike rate) (F) for OR59b and OR22a neurons during stimulation with different concentrations of methyl butyrate. Boxplots show the median and interquartile range across 14 OR59b and 12 OR22a neurons (same colored points represent same neuron). Numbers at the top indicate the median. Stars indicate significant difference between medians of neighboring concentrations (*p < 0.05, **p < 0.01, ***p < 0.001). See also Figures S1 and S2.

To determine the speed and temporal precision of odorant-evoked spikes in olfactory receptor neurons, we recorded from pairs of *Drosophila* olfactory receptor neurons that express the same olfactory receptor (neurons expressing the receptor OR59b and neurons expressing OR22a, Figure 1A; original data are available in Data S1). To mimic the intermittent and rapid odorant stimuli that insects encounter when flying through an odor plume (Celani et al., 2014, Farrell et al., 2002, Huston et al., 2015, Murlis, 1992, Riffell et al., 2014, van Breugel and Dickinson, 2014), we presented brief odorant pulses with rapid changes in concentration (Figure 1A).

### First Spike Timing Varies Over a Wider Concentration Range than Spike Rate

Odorant-evoked spikes were tightly locked to the onset of odorant pulses (Figures 1B, 1C, S1B, and S1C), with first spike latencies ranging from 18 to 55 ms for low concentrations and 3 to 4.4 ms for high concentrations (median latencies for 10 repeated stimulations; Figures 1D and S2A). First spike latencies were temporally precise across trials, with an average SD (trial-to-trial jitter) of 4.36–106 ms for low odorant concentrations and 0.19–0.49 ms for high concentrations (Figures 1E and S2B). The neuron-to-neuron jitter was similar to the trial-to-trial jitter and ranged from 4.82 to 107 ms for low concentrations and 0.2–0.91 ms for high concentrations (Figure S2C). First spike latency, jitter, and spike rate varied with odorant concentration, and first spike latencies varied over a wider concentration range than spike rates (represented as minimum interspike or first-to-second spike interval; Figures 1D–1F and S2).

The concentration dependency of response latencies has been previously reported in insects (Martelli et al., 2013) and vertebrates (Duchamp-Viret et al., 1999, Getchell and Shepherd, 1978). However, the minimum odorant-evoked first spike latency of 3 ms that we found (Figures 1 and S2A) is shorter than previously reported for insect olfactory receptor neurons (10–30 ms [De Bruyne et al., 2001, Schneider et al., 1964]) or vertebrate olfactory receptor neurons (50 ms [Firestein et al., 1990]). Likewise, the minimum first spike latency jitter of 0.19 ms (Figures 1 and S2B) is more than one order of magnitude smaller than previously reported for insect olfactory receptor neurons (7 ms jitter [Jeanne and Wilson, 2015]) or for vertebrate olfactory receptor neurons (12 ms jitter [Shusterman et al., 2011]). Such a high spike timing precision is comparable with the precision of insect photoreceptor cells (0.1 ms jitter [Tatler et al., 2000]) or insect auditory receptor cells (0.16 ms jitter [Rokem et al., 2005]).

Our finding of shorter response latencies and higher spike timing precision than in previous studies could be explained by the short stimulus rise time (5% to 95% within 3.6 ms) of our olfactory stimulator, which is one to two orders of magnitude shorter than the stimulus rise time of commonly used olfactory stimulators (Martelli et al., 2013, Raiser et al., 2016), and by the fact that response strength and precision of insect olfactory receptor neurons increase with decreasing stimulus rise time (Kim et al., 2011, Martelli et al., 2013, Nagel and Wilson, 2011, Tichy et al., 2005, Tichy et al., 2016).

### Anatomical Convergence Allows Rapid Odorant Onset Detection

**Figure 2:**
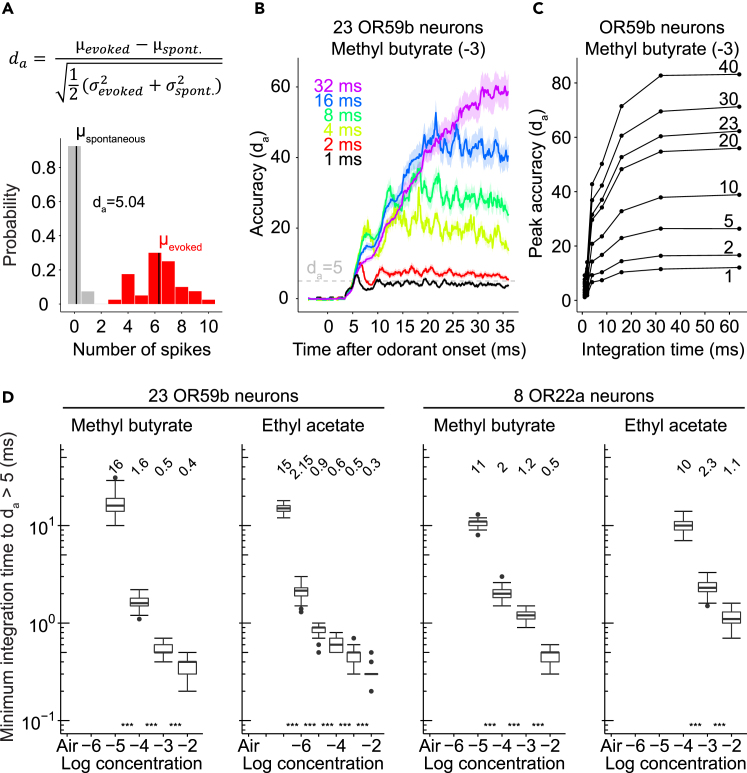
Anatomical Convergence Allows Rapid Odorant Onset Detection (A) Odorant detection accuracy (d_a_) was measured as the difference between the means of odorant-evoked (red) and spontaneous spike counts (gray), normalized to the SD of both. Distributions of spontaneously generated spikes and odorant-evoked spikes of a pool of 23 OR59b neurons (methyl butyrate at a concentration of 10^−3^, integration time [1 ms], μ = mean, σ = SD). (B) Detection accuracy (d_a_, mean ± SD) computed for randomly selected pools of 23 OR59b neurons for different integration times (1–32 ms) during stimulation with methyl butyrate at a concentration of 10^−3^. (C) Peak detection accuracy (d_a_) for different integration times (1, 2, 4, 8, 16, 32, 64 ms) and OR59b neuron pool sizes (1–40). (D) Minimum integration time to exceed a detection accuracy of five during stimulation with different concentrations of methyl butyrate or ethyl acetate. Boxplots show the median and interquartile range for 50 repetitions, each computed from 40 random combinations of 23 OR59b or 8 OR22a neurons. At missing entries, the detection accuracy did not exceed five within an integration time window of 40 ms. Numbers at the top indicate the median. Stars indicate statistical difference between medians of neighboring concentrations (***p < 0.001). See also Figures S3 and S4.

The detection of an odorant requires separating odorant-evoked from spontaneous spikes. We quantified this separation as detection accuracy (d_a_), which is the difference between the mean number of odorant-evoked and spontaneous spikes divided by their root-mean-square SD (Jeanne and Wilson, 2015, Simpson and Fitter, 1973) (Figure 2A and Transparent Methods). We calculated the detection accuracy for a pool of 23 OR59b and of 8 OR22a neurons, because in female flies, about 23 OR59b and 8 OR22a neurons coalesce in different glomeruli (DM4 and DM2) of the antennal lobe (Grabe et al., 2016) and because all receptor neurons converge onto all uniglomerular projection neurons (Kazama and Wilson, 2009). Detection accuracy increased with the spike integration time window (integration time) (Figures 2B and S3) and with the number of receptor neurons (Figures 2C and S4). For a given odorant concentration, detection accuracy first increased independently of integration time (first 1 to 2 ms) and continued increasing with longer integration times (Figures 2B and S3A).

To estimate how odorant detection speed depends on concentration, we determined the minimum integration time to reach a threshold detection accuracy above five (Figure 2A), which corresponds to a false-positive rate of less than 1% for separating odorant-evoked from spontaneous spikes (see Transparent Methods). This detection accuracy threshold was reached with integration times between 10 and 16 ms already at the lowest odorant concentrations, and with integration times of less than 3 ms at intermediate concentrations. Importantly, at all concentrations this detection accuracy threshold was reached with an integration time that is shorter than the corresponding minimum interspike interval (1/spike rate) (Figures 2D, 1F, and S2). This indicates that the first odorant-evoked spikes across a population of 23 OR59b or 8 OR22a neurons are sufficient for encoding the onset of an odorant, even at low odorant concentrations.

### Encoding of Odorant Identity with Spike Latencies

**Figure 3:**
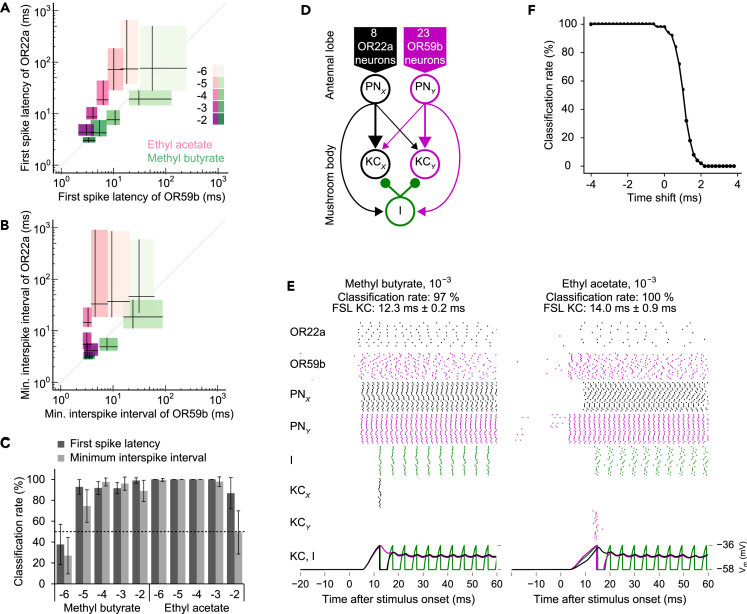
Relative Spike Latencies Allow Concentration-Invariant Rank Order Encoding of Odorant Identity (A) First spike latencies of OR59b versus OR22a neurons for different concentrations of methyl butyrate and ethyl acetate (lines, median; boxes, 5^th^ to 95^th^ percentile range). (B) Same as in (A) but for minimum interspike intervals. (C) Classification of odorants based on the differences in first spike latencies or minimum interspike intervals between OR22a and OR59b neurons (mean ± SD). A single classification threshold was used across odorants and concentrations. Dashed line shows 50% chance level. (D) Simplified spiking neural network model that is sensitive to the relative first spike latencies across receptor neuron types. Eight OR22a neurons form excitatory synapses with projection neuron PN_*X*_ and 23 OR59b neurons with PN_*Y*_. PNs form excitatory synapses with two Kenyon cells (KC_*X*_ and KC_*Y*_) and with an inhibitory neuron (I), which provides feedforward inhibition to both KCs. Individual PN-KC synapses differ in weight (arrow thickness reflects synaptic weights of PN-KC and PN-I synapses). (E) Simulation results. From top to bottom: Recorded spikes in 8 OR22a and 23 OR59b neurons, simulated spikes in PN_*X*_ and PN_*Y*_, I, KC_*X*_ and KC_*Y*_, and simulated membrane potentials in I (green), KC_*X*_ (black), and KC_*Y*_ (magenta). Receptor neuron spike raster plots and membrane potentials show the first simulation, all other spike raster result from the first 20 simulations. Classification rate is the percentage of correct classifications during 100 simulation runs for each odorant. Odorant classification was correct when methyl butyrate induced a spike in KC_*X*_ but not in KC_*Y*_ and when ethyl acetate induced a spike in KC_*Y*_ but not in KC_*X*_. Left: Methyl butyrate (concentration 10^−3^) first activates OR22a and then OR59b neurons. Since the weight of the PN_*X*_-KC_*X*_ synapse is larger than the weight of the PN_*X*_-KC_*Y*_ synapse, the PN_*X*_-driven inhibition prevents KC_*Y*_ from reaching its spiking threshold. Right: Ethyl acetate first activates OR59b and then OR22a and induces spikes in KC_*Y*_ but not in KC_*X*_. FSL KC, first spike latency of Kenyon cells. (F) To test the sensitivity of the network to the relative timing of spikes, the recorded spike trains (during stimulation with methyl butyrate, 10^−3^) of all 8 OR22a neurons were shifted in time (from −4 to 4 ms relative to their original occurrence) in 0.2 ms steps while keeping the 23 OR59b spike trains unchanged. Classification rate (classified as methyl butyrate) decreases when spikes from OR22a neurons are shifted by 1–2 ms. See also Figure S5 and Audios S1, S2, S3, S4, S5, S6, S7, S8, S9, S10, and S11.

First spike latencies (relative to odorant arrival at the antenna) were temporally precise and odorant-specific (Figures 1D and S2A). Could the olfactory system use first spike latencies to encode odorant identity? First spike latencies may seem unsuitable for encoding odors, because the decoder does not know when the stimulus started. However, the olfactory system could use the difference of first spike latencies across different receptor neuron types (relative spike latencies), as has been demonstrated for other sensory systems (Gollisch and Meister, 2008, Thomson and Kristan, 2006, Thorpe et al., 2001). To test whether relative spike latencies across receptor neuron types could encode odorant identity, we compared the across-neuron pattern of first spike latencies and spike rates (minimum interspike intervals) (Figures 3A and 3B). OR59b neurons responded faster (shorter first spike latencies) and with higher spike rates (shorter inter spike intervals) to ethyl acetate than OR22a neurons, whereas OR22a neurons responded slightly faster and with higher spike rates to methyl butyrate than OR59b neurons. Both the difference in first spike latencies and the difference in interspike intervals (1/rate) between the two receptor neuron types allowed concentration-invariant classification of odorant identity across four to five orders of magnitude in concentration using a single classification threshold for all concentrations (0.76 ms difference in first spike latencies and 0.77 ms difference in interspike intervals) (Figure 3C and see Audios S1, S2, S3, S4, S5, S6, S7, S8, S9, S10, and S11 for audible examples of odorant-evoked spikes across 23 OR59b and 8 OR22a neurons).

Based on these results, we propose a coding scheme in which the first wave of odorant-evoked spikes across the population of the first responding olfactory receptor neuron type encodes the onset of an odorant (spikes are almost in synchrony, due to low jitter, Figures 1C and 1E), whereas the rank order (relative spike latencies, Figures 3A and 3C) of the subsequently responding olfactory receptor neuron types encodes the identity of that odorant in a concentration-invariant manner. Rank order codes for odorant identity have been previously proposed for insects (Brill et al., 2013, Krofczik et al., 2009, Martelli et al., 2013) and vertebrates (Junek et al., 2010, Schaefer and Margrie, 2012, Spors, 2006), although at slower timescales.

To test whether the insect olfactory system could use the millisecond short differences in spike latencies across olfactory receptor neurons for odor coding, we built a spiking neural network for odorant classification, which contains network motifs of the second layer of olfactory processing, the mushroom body (Figure 3D). All neurons were simulated with a leaky integrate-and-fire model, using the same approach as Jeanne and Wilson (Jeanne and Wilson, 2015). As input to the model we randomly selected 8 OR22a and 23 OR59b neurons from the pool of our recordings. OR22a and OR59b neurons converge onto different projection neurons (PN_*X*_ and PN_*Y*_) (Grabe et al., 2016, Kazama and Wilson, 2009), which form excitatory synapses with different weights onto mushroom body intrinsic Kenyon cells (KC_*X*_ and KC_*Y*_) (Gruntman and Turner, 2013). In addition, projection neurons provide feedforward inhibition onto Kenyon cells via an inhibitory neuron (I). Such an inhibitory feedforward circuit via a GABAergic neuron exists in honey bees within the mushroom input region (calyx) (Ganeshina and Menzel, 2001), and it might also exist within the mushroom body calyx of other insects (e.g., *Drosophila* [Lei et al., 2013, Lin et al., 2014], locusts [Leitch and Laurent, 1996, Papadopoulou et al., 2011]). Note that for the sake of simplicity and as a proof of principle, we ignored several properties of the *Drosophila* olfactory systems (see Transparent Methods).

This network resembles a network model by Thorpe and colleagues (Thorpe et al., 2001), which is sensitive to the rank order of spike arrival times from different inputs, and it resembles a (recently refuted) model by Assisi and colleagues (Assisi et al., 2007, Gupta and Stopfer, 2012) in which Kenyon cells' integration time is shortened through feedforward inhibition. Notably, a similar circuit motif for the required rapid feedforward inhibition (Thorpe et al., 2001) also exists in the second layer of the vertebrate olfactory system, the olfactory cortex (Stokes and Isaacson, 2010). In our model, the depolarization of Kenyon cells depends on the different synaptic weights from projection neurons and is strongest when projection neurons are activated in the order of their synaptic weights (Figures 3E and S5). This is because feedforward inhibition increases with the number of inputs (see Thorpe et al. (2001) for a detailed analysis of a similar rank-order-sensitive network). Note that since inputs from PN_*Y*_ are weighted lower than inputs from PN_*X*_, KC_*X*_ can respond even when PN_*Y*_ responds shortly before PN_*X*_.

Using this rank-order-sensitive network, classification of the two odorants, based on the experimentally measured spike trains in OR22a and OR59b neurons, was rapid (within 10 and 28 ms after odorant arrival) and reliable (between 78% and 100% correct classification) at concentrations between 10^−4^ and 10^−2^ (Figures 3E and S5). To test the rank-order sensitivity of the model independently of spike rate differences, we repeated the simulations with artificially introduced time shifts in the experimentally measured spike trains of one olfactory receptor neuron type while leaving the other at its original time (Figure 3F). Odorant classification was correct, when the rank order was retained by the time shift, showing that the model is sensitive for the rank order of olfactory receptor neuron inputs. The exact time shift at which the classification decreases depends on the synaptic weights between the PN and KC, as stronger weights lead to a faster response in the respective KC. This simplified network model of the mushroom body demonstrates that odorant-specific response latencies across olfactory receptor neuron types, together with rapid feedforward inhibition, allow encoding of odorant identity with relative spike latencies.

Rapid odorant detection is likely not unique to *Drosophila* receptor neurons, as rapid odorant-evoked antennal responses occur in other insects (Szyszka et al., 2014), all of which face the challenge of detecting odorants when moving through turbulent air (Celani et al., 2014, Murlis, 1992). The need for speed could have promoted the evolution of pore tubules that accelerate odorant diffusion toward olfactory receptors (Maitani et al., 2010, Steinbrecht, 1997) and of rapid ligand-gated ionotropic olfactory receptors (Getahun et al., 2012, Sato et al., 2008, Silbering and Benton, 2010, Wicher et al., 2008) that allow faster stimulus transduction than metabotropic receptors (house fly photoreceptors, which are the fastest known metabotropic receptors, have a minimum response latency of 12 ms [Howard et al., 1984]).

Insect olfaction is similar to vertebrate audition in that both senses continuously sample the temporal structure of stimuli. The vertebrate auditory system relies on precise spike timing when it uses stimulus onset asynchrony and interaural time differences to separate and to localize sound sources (Knudsen and Konishi, 1979, Rasch, 1978). Analogous to the vertebrate auditory system, the insect olfactory system can use millisecond short stimulus onset asynchrony for odor source separation (Baker et al., 1998, Szyszka et al., 2012), and it may use bilateral time differences for source localization during active sampling of odorant gradients (Borst and Heisenberg, 1982, Duistermars et al., 2009, Gaudry et al., 2013, Hangartner, 1967, Louis et al., 2008). In addition to supporting odor source separation and localization, the high temporal precision of odor-evoked spikes across olfactory receptor neurons could allow the insect olfactory system to use a rapid, spike timing-based code for odorant identity.

## Methods

All methods can be found in the accompanying Transparent Methods supplemental file.

## Supplemental Information

Document S1. Transparent Methods, Figures S1–S5, and Table S1

Audio S1. Sound of Blank Air, Related to Figure 3C Odorant-evoked spikes across 8 OR22a neurons (left channel) and 23 OR59b neurons (right channel) during ten stimulations with blank air (first stimulus after 1.75 s, 2 s inter stimulus interval). A total of 8 (OR22a) or 23 (OR59b) randomly selected, 2-s-long spike trains were summed up into one trace and were convolved with an exponential kernel of tau = 0.5 ms (OR22a) or 0.2 ms (OR59b) and an amplitude of 0.15. This was repeated ten times and the results were concatenated along the time axis.

Audio S2. Sound of Methyl Butyrate (10^−6^), Related to Figure 3C Same as Audio S1 but during stimulation with methyl butyrate at a concentration of 10^−6^.

Audio S3. Sound of Methyl Butyrate (10^−5^), Related to Figure 3C Same as Audio S1 but during stimulation with methyl butyrate at a concentration of 10^−5^.

Audio S4. Sound of Methyl Butyrate (10^−4^), Related to Figure 3C Same as Audio S1 but during stimulation with methyl butyrate at a concentration of 10^−4^.

Audio S5. Sound of Methyl Butyrate (10^−3^), Related to Figure 3C Same as Audio S1 but during stimulation with methyl butyrate at a concentration of 10^−3^.

Audio S6. Sound of Methyl Butyrate (10^−2^), Related to Figure 3C Same as Audio S1 but during stimulation with methyl butyrate at a concentration of 10^-−2^.

Audio S7. Sound of Ethyl Acetate (10^−6^), Related to Figure 3C Same as Audio S1 but during stimulation with ethyl acetate at a concentration of 10^−6^.

Audio S8. Sound of Ethyl Acetate (10^−5^), Related to Figure 3C Same as Audio S1 but during stimulation with ethyl acetate at a concentration of 10^−5^.

Audio S9. Sound of Ethyl Acetate (10^−4^), Related to Figure 3C Same as Audio S1 but during stimulation with ethyl acetate at a concentration of 10^−4^.

Audio S10. Sound of Ethyl Acetate (10^−3^), Related to Figure 3C Same as Audio S1 but during stimulation with ethyl acetate at a concentration of 10^−3^.

Audio S11. Sound of Ethyl Acetate (10^−^^2^), Related to Figure 3C Same as Audio S1 but during stimulation with ethyl acetate at a concentration of 10^−2^.

Data S1. Original Spike Data, Related to Figure 1 The file contains 3020 rows. The first six values in every row contain the following information: 1) Date of the recording (e.g., **X160712**) 2) Fly number respective to other flies of the same day (e.g., **1a**) 3) Sensillum type (**ab3** corresponding to OR22a, **ab2** corresponding to OR59b) 4) Odorant and concentration (log dilution). **MEBE (methyl butyrate)**, **EtAc (ethyl acetate)**, and **Air** (odorless blank control). Concentrations range from 10^−2^ to 10^−6^, except for ab2_EtAc, were in some measurements concentrations range from 10^−2^ to 10^−8^. 5) Recording channel. Columns with identical names that only differ in **U1/U2** contain spike trains of the two simultaneously recorded neurons. 6) Trial number. Each stimulus was presented 10 times and for every recording **T** ranges from **1** to **10**. All other values in each row are spike times relative to the stimulus trigger in seconds. Recordings start at 3.005 s before the trigger to 0.25 s after the trigger. Note that the odorant arrives at the antenna 4.75 ms after the trigger, and that the spike times are the peak time minus 0.84 ms (corresponding to the onset of the window used for spike sorting). To get the peak spike times relative to odorant arrival at the antenna, add 5.59 ms (4.75 + 0.84 ms) to the spike times.
