# Supplementary material for: High Precision of Spike Timing across Olfactory Receptor Neurons Allows Rapid Odor Coding in Drosophila
Source: iScience. 2018 May 17;4:76–83. doi: 10.1016/j.isci.2018.05.009 (PMC6147046; doi:10.1016/j.isci.2018.05.009)
Supplement: Document S1. Transparent Methods, Figures S1–S5, and Table S1 [file mmc1.pdf]

ISCI, Volume *4*

## Supplemental Information

# High Precision of Spike Timing across Olfactory Receptor Neurons Allows Rapid Odor Coding in *Drosophila*

**Alexander Egea-Weiss, Alpha Renner, Christoph J. Kleineidam, and Paul Szyszka**

## Supplemental Figures

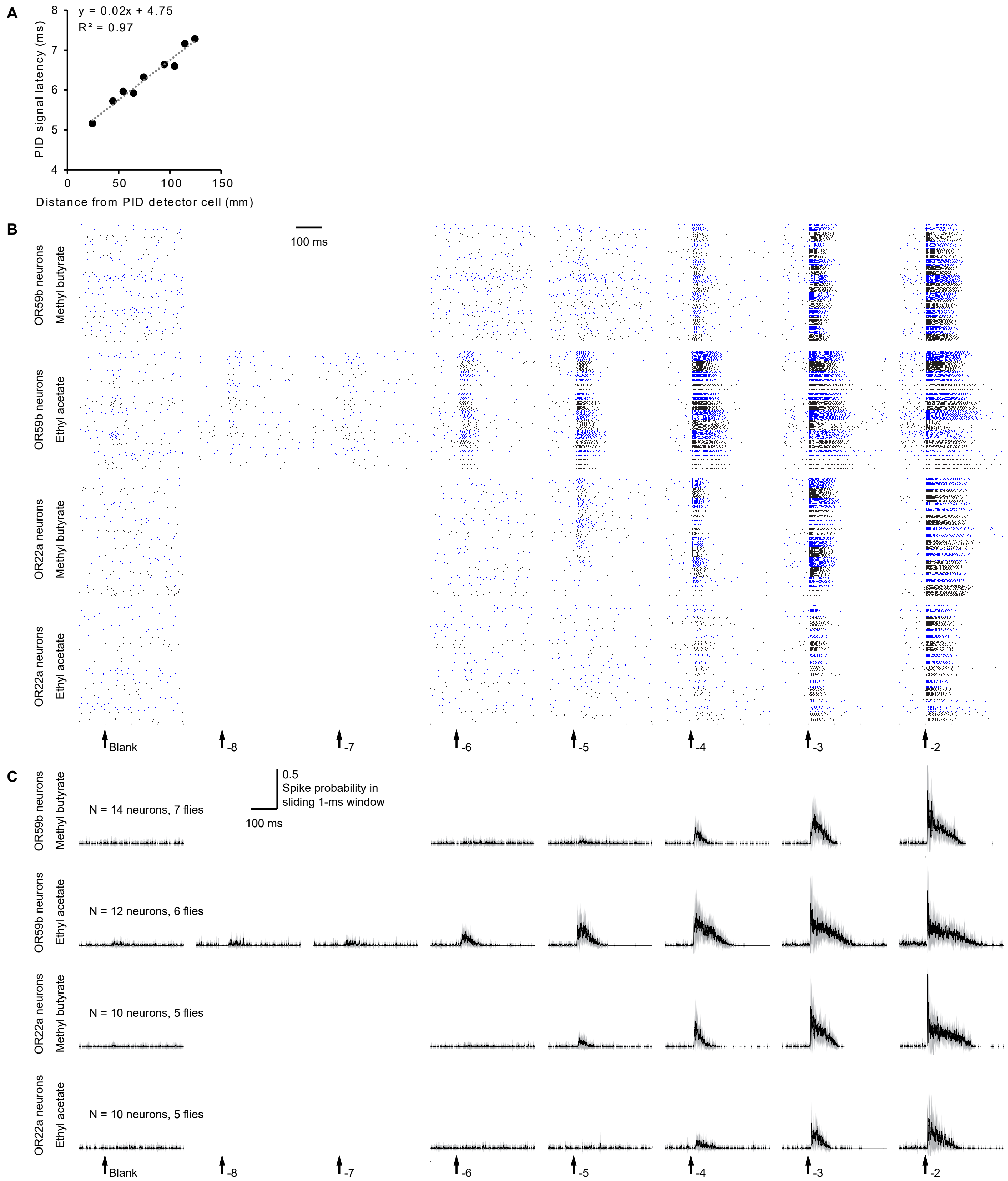


**Figure S1. Reproducible, concentration-dependent spike responses across neurons and flies, Related to Figure 1 and Transparent Methods.** (A) Stimulus latency was determined with a photoionization detector (PID, miniPID, Aurora) and ethyl acetate ($10^{-2}$) as tracer substance. A 125 mm long glass tube served as inlet of the PID. The glass tube ended at the detector cell. The length of the glass tube was gradually shortened, while measuring the PID signal latency. The PID signal latency decreased linearly ($R^2 = 0.97$, linear regression) with the decrease of the distance to the detector cell. The 4.75 ms stimulus latency was extrapolated as the time at which the distance to the detector cell was 0 mm (intercept with the y-axis). (B) Raster plot of simultaneously recorded spikes in pairs of OR59b and OR22a neurons during 10 blank or odorant stimuli (methyl butyrate or ethyl acetate) at different concentrations ($10^{-8}$ to $10^{-2}$). Adjacent blue and black points represent spikes from simultaneously recorded neuron pairs. (C) Same data as in (B) presented as spiking probability during sliding 1 ms windows (mean ± SD).

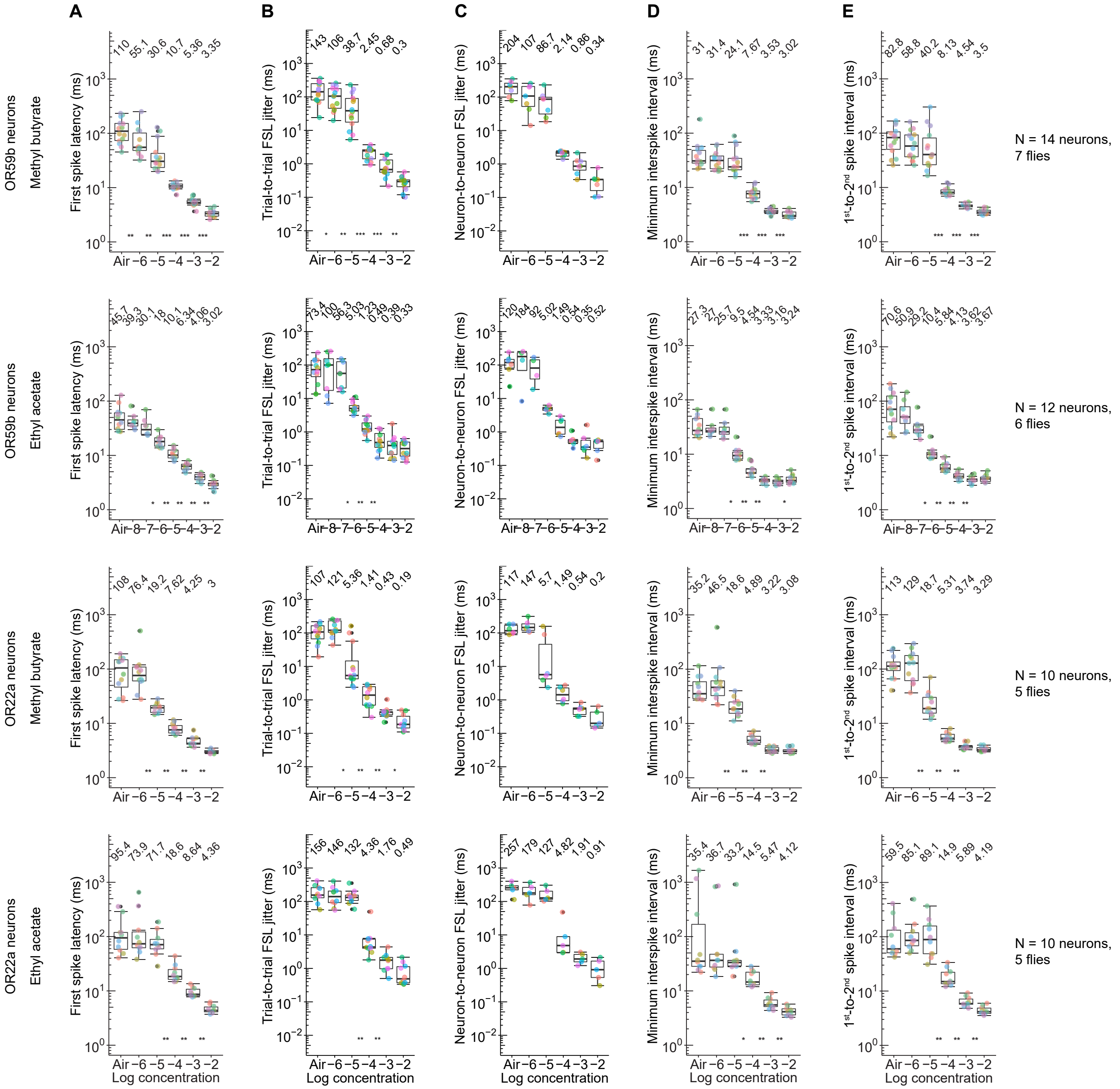

**Figure S2. First spike timing varies over a wider concentration range than spike rate, Related to Figure 1.**
(A – E) First spike latencies (A), trial-to-trial jitter of first spike latencies (FSL) (B), neuron-to-neuron jitter of first spike latencies (C), minimum interspike intervals (1/maximum rate) (D), and interval between the first and second spikes after odorant onset (E) for OR59b and OR22a neurons during stimulation with different concentrations of methyl butyrate or ethyl acetate. Boxplots show the median and interquartile range across neurons (colored points, same color represents same neuron). Numbers at the top indicate the median. Stars indicate statistical difference between medians of neighboring concentrations (* p<0.05, ** p<0.01, *** p<0.001).

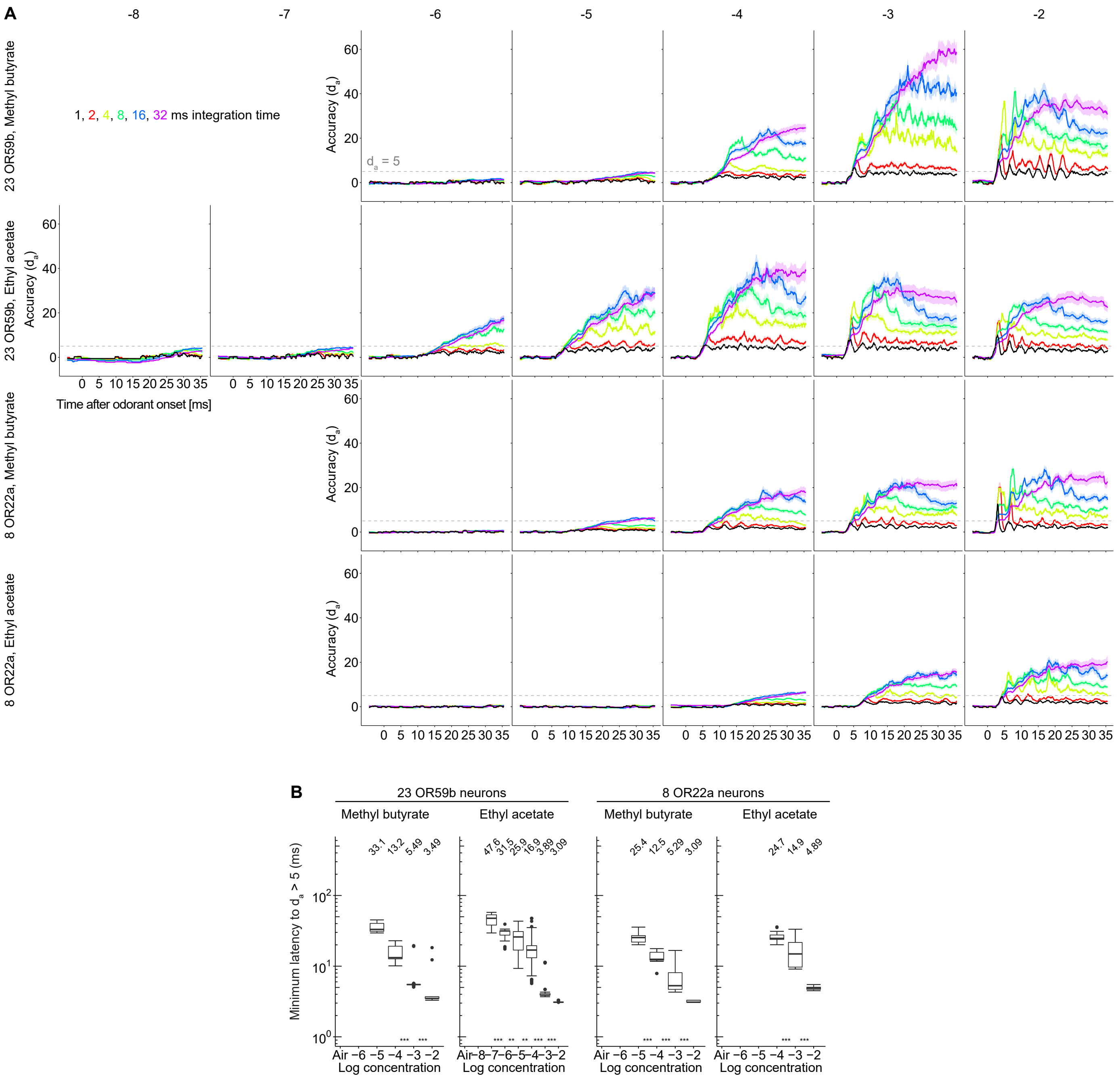


**Figure S3. Stimulus detection accuracy depends on odorant concentration and integration time, Related to Figure 2.**

(A) Stimulus detection accuracy (da, mean ± SD) computed for a simulated pool of 23 OR59b and 8 OR22a neurons for different integration time windows (1 – 32 ms) during stimulation with methyl butyrate and ethyl acetate at different concentrations ($10^{-8}$ – $10^{-2}$).(B) Minimum latency (first spike latency + same integration time windows as in Figure 2D) to reach a detection accuracy above 5 during stimulation with different concentrations of methyl butyrate and ethyl acetate in 23 OR59b and 8 OR22a neurons. At missing entries the detection accuracy did not exceed 5 within an integration time window of 40 ms. Numbers at the top indicate the median. Stars indicate statistical difference between medians of neighboring concentrations (** p<0.01, *** p<0.001).

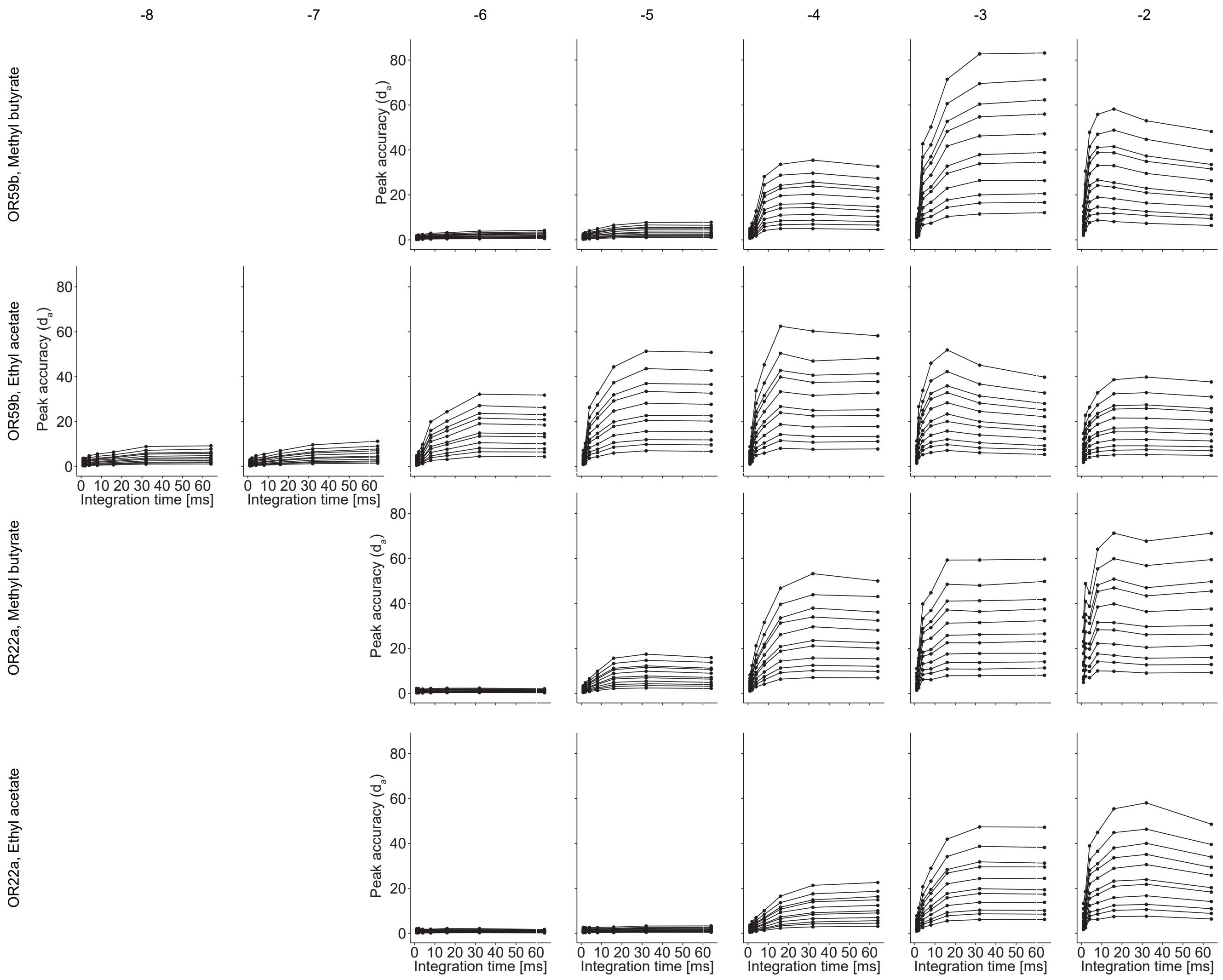


**Figure S4. Peak detection accuracy depends on odorant concentration, neuron number and integration time window, Related to Figure 2.** Peak stimulus detection accuracy (da) for different integration time windows (1 – 64 ms) and number of neurons (1, 2, 3, 5, 8, 10, 15, 20, 23, 30, 40) for stimulation with methyl butyrate and ethyl acetate at different concentrations ($10^{-8}$ – $10^{-2}$).

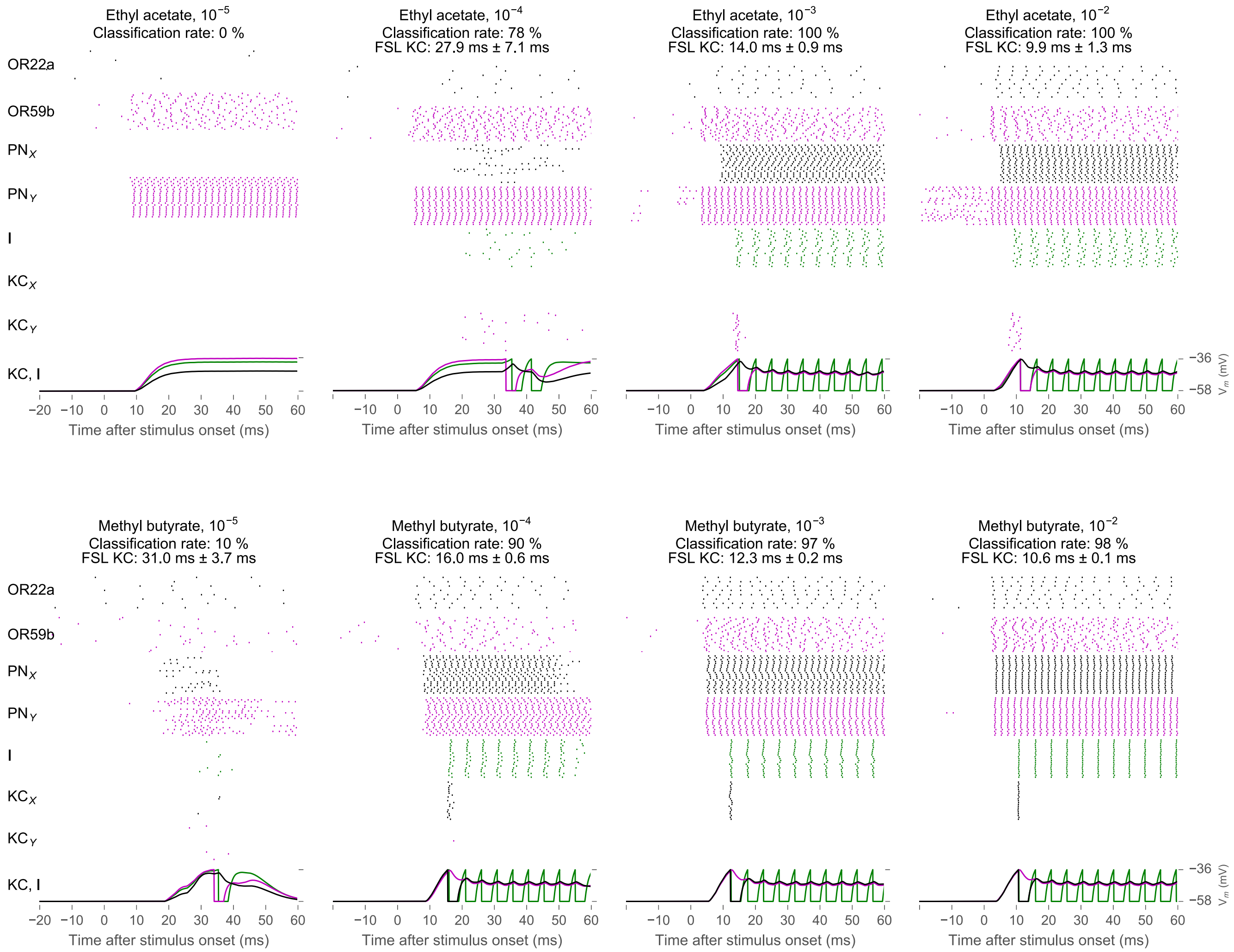


**Figure S5. Simulation results from the spiking neural network model for odorant classification, Related to Figure 3.**

In each panel, from top to bottom: Recorded spikes in 8 OR22a and 23 OR59b neurons, simulated spikes in PNX and PNY, I, KCX and KCY, and simulated membrane potentials in I (green), $KC_X$ (black) and $KC_Y$ (magenta). Left: Methyl butyrate (concentration $10^{-3}$) first activates OR22a neurons and then OR59b neurons. Right: Ethyl acetate first activates OR59b neurons and then OR22a neurons and induces spikes in $KC_Y$ but not in $KC_X$. Odorant classification was correct when methyl butyrate induced a spike response in $KC_X$ but not in $KC_Y$ and when ethyl acetate induced a spike response in $KC_Y$ but not in $KC_X$. Classification rate was calculated as the percentage of correct classifications during 100 simulation runs for each odorant. Receptor neuron spike raster and membrane potentials result from the first simulation, all other spike raster result from the first 20 simulations. Note that in this model classification only works reliably for higher concentrations, because in order to be activated, each KC requires inputs from both PNs in the right order.

**Supplemental Table**

**Table S1. Parameters of model neurons, Related to Figure 3**. Parameters of PNs and PN-ORN synapses are the same or close to the ones from (Jeanne and Wilson, 2015; Nagel and Wilson, 2011). Parameters of KCs and PN-KC synapses are the same or close to the ones from (Turner et al., 2008). In (Turner et al., 2008) values were given per unit, which explains the large difference to the PN parameters. Note that for the simulation only relative values within a neuron and its pre-synapses matter. For the inhibitory neuron (I) we took the same parameters as for KC, due to a lack of published data. Free parameters that were tuned to achieve a good classification rate were the synaptic weights (J) PN-KC, PN-I and I-KC.

| **Neuron parameter** | **PN** | **KC, I** |
|---|---|---|
| $G_l$ (leak conductance) | 4.35 nS | 90 µS |
| $V_{res}$ (reset and leak reversal potential) | -55 mV | -58 mV |
| $V_{thr}$ (threshold potential) | -40 mV | -36 mV |
| $C_m$ (membrane capacitance) | 0.135 pF | 1µF |
| $t_{ref}$ (refractory period) | 2 ms | 3 ms |

<table>
<tr><th>Synapse parameter</th><th>ORN-PN</th><th>PN-KC, PN-I</th><th>I-KC</th></tr>
<tr><td>$\tau$ (EPSG time scale)</td><td>3 ms</td><td>3 ms</td><td>3 ms</td></tr>
<tr><td>$V_{rev}$ (reversal potential)</td><td>0 mV</td><td>0 mV</td><td>-65 mV</td></tr>
<tr><td rowspan="5">$J$ (EPSG peak amplitude)</td><td rowspan="5">0.27 nS</td><td>$J_{XX}$ = 70 µS</td><td rowspan="5">400 µS</td></tr>
<tr><td>$J_{YY}$ = 0.81 * $J_{XX}$</td></tr>
<tr><td>$J_{XY}$ = 0.5 * $J_{XX}$</td></tr>
<tr><td>$J_{YX}$ = 0.5 * $J_{YY}$</td></tr>
<tr><td>$J_{PN\text{-}I}$ = ($J_{XX}$ + $J_{YY}$ + $J_{XY}$ + $J_{YX}$)/4</td></tr>
</table>

## Transparent Methods

### Fly stock and maintenance

All experiments were done with 2 – 7 days old, female wild-type Canton-S *Drosophila melanogaster*. Flies were raised at 25 °C on standard *Drosophila* medium, with a 12/12 h day/night cycle.

### Fly preparation

Electrophysiological recordings of olfactory receptor neurons were performed on large basiconic ab2 sensilla (OR59b neurons) and ab3 sensilla (OR22a neurons) of the left antenna. The fly was fixed in a 200 µL plastic pipette tip with a piece of cotton, and the left antenna was glued with low melting wax (1:1:1 mixture of n-eicosan, myristic acid and dental wax (Deiberit 502; Dr. Böhme und Schöps Dental)) to gain access to the medial side. The right antenna was covered with low melting wax. The two recording electrodes were positioned with micromanipulators (Kleindieck) and inserted into two sensilla of the same type under a microscope (Axioplan, Zeiss) equipped with a 50x objective (LMPlanFl 50x/0.50, Olympus). The identity of the large basiconic ab2 and ab3 sensilla was determined by their response to a panel of 5 diagnostic odorants (methyl butyrate, isobutyl acetate, methyl acetate, 2-butanone and ethyl hexanoate at a concentration of $10^{-3}$, all from Sigma Aldrich) which were applied as 500 ms long pulses via a multichannel odorant delivery device (Szyszka et al., 2011). Deflections in the sensillum potential and increase in spike rate were taken as excitatory responses to a given odorant. Sensilla showing moderate responses to 2-butanone and strong responses to methyl butyrate and methyl acetate were identified as ab2 sensilla, whereas sensilla responding strongly to methyl butyrate, isobutyl acetate and ethyl hexanoate were identified as ab3 sensilla (Database of Odorant Responses, (Münch and Galizia, 2016)).

### Electrophysiology

Tungsten wires (diameter = 0.1mm) that were electrolytically sharpened with AC-current in a 0.5 M KOH solution were used as recording and reference electrodes. Two recording electrodes were inserted in two sensilla of the same type, and the reference electrode was inserted into the complex eye. Lubricant gel (Hydro Sensitiv Gel, Ritex) was applied to the contact zone between the reference electrode and the eye in order to increase electric conductance and to keep the eye from drying out. Signals of the recording electrodes were differentially amplified against the reference electrode using 1000x gain and a 1.0 Hz and 8.0 kHz bandpass filter (MA 103 preamplifier and MA 102 4-channel amplifier, Universität zu Köln). Noise from the powerline was reduced with a Hum Bug (Quest Scientific) and signals were digitized with a Micro 3 1401 (CED). Stimulation and recordings were controlled with Spike2 software (version 7.03 CED).

### Odorant delivery

Methyl butyrate and ethyl acetate (Sigma-Aldrich) were diluted in 10 mL mineral oil (Sigma-Aldrich) in 120-mL headspace bottles. During experiments, the odorant solutions were continuously stirred with a magnetic stirrer to create an equilibrated headspace. Odorant solutions were freshly made every month (corresponds to approximately 10 stimulations). Fast odorant pulses were applied by opening a 3-way solenoid valve for 3 ms (LFAA1200118H; Lee) (Szyszka et al., 2014). The flow rate was adjusted to 190 mL per minute (charcoal-filtered air, non-humidified, digital air pressure control (35898; Analyt-MTC) and flow control (rotameter, 112-02GL; Analyt-MTC)). The inner diameter of the Teflon tube at the stimulator outlet was 1 mm, the distance between the stimulator outlet and the antenna was 1 mm, and the distance between antenna and the gate of the valve was 16.5 mm. The gate of the valve starts moving around 0.4 ms after the electrical trigger signal that switches the valve (Szyszka et al., 2014). Assuming a laminar airflow and no pressure changes, the odorant would arrive 4.5 ms after the trigger signal that operated the valve (0.4-ms valve delay plus 4.1-ms air travel time).

We determined the actual stimulus latency (time between the valve trigger and the arrival of the odorant at the antenna) with a photoionization detector (PID, miniPID, Aurora), using ethyl acetate ($10^{-2}$) as tracer substance. The inlet needle of the PID was replaced by a 125 mm long glass tube that ended at the PID detector cell, and the PID pump was set to the highest speed. The length of the glass tube was gradually shortened, while measuring the PID signal latency for ten stimulus repetitions. The PID signal latency was determined by first averaging the PID signal over the ten stimulus repetitions in order to decrease noise, and then moving backwards from the time where the response is 5 standard deviations above baseline until the PID signal reached the baseline (mean PID signal over first 4 ms after valve trigger). The PID signal latency decreased linearly ($R^2$ = 0.97, linear regression) with decreasing

distance to the PID detection cell (Figure S1A). The stimulus latency of 4.75 ms was extrapolated, and corresponds to the time at which the distance to the detection cell would be 0 mm. This value was subtracted from all spike latencies.

This olfactory stimulator generates fast changes in odorant concentration (rise time 5%-to-95% within 3.6 ms, Figure 1A), and is one to two orders of magnitude faster than commonly used olfactory stimulators (Martelli et al., 2013). We delivered sequences of 10 odorant pulses by opening the valve for 3 ms, with an interpulse interval of 5 s. We applied series of odorant pulses with different concentrations ($10^{-8}$, $10^{-7}$, $10^{-6}$, $10^{-5}$, $10^{-4}$, $10^{-3}$, and $10^{-2}$). Recordings started with the application of a blank stimulus (empty headspace bottle), followed by the odorant stimuli with rising odor concentration. After a complete sensillum recording the valve was flushed with air over night, in order to remove residual odorants in the valve.

To determine the rise time of the odorant stimulus (Figure 1A), we replaced the original stainless steel inlet needle of the PID by a 24.5 mm long glass needle in order to reduce low pass filtering of the odorant due to absorption at the inlet needle.

**Data Analysis**

**Spike sorting**: Spikes were sorted with Spike2 and then reviewed manually. The data was analyzed with custom scripts in R (version 3.1.1). The spike time was defined as time when the spike amplitude reached its maximum. Original spike time data is available in Data S1.

**First spike latency**: The time between the arrival of the stimulus at the fly antenna and the first recorded spike after stimulus arrival was taken as the first spike latency. In cases where the stimulus did not elicit a response, the first spike latency is the latency of the first spontaneously generated spike after stimulus onset. The colored points in Figure 2A show the median first spike latencies over 10 responses for each recorded neuron.

**Spike rate**: In order to facilitate the comparison between spike rate and first spike latency, we used the inverse spike rate, the interspike interval (time between two spikes n and n + 1). The minimum interspike interval was determined for a 500 ms window after the first spike. The colored points in Figure 2C show the median interspike interval over 10 responses for each recorded neuron.

**Trial-to-trial jitter of first spike latency**: The trial-to-trial jitter of the first spike latency was quantified as the standard deviation of the first spike latencies across 10 stimulus repetitions.

**Neuron-to-neuron jitter of first spike latency**: The neuron-to-neuron jitter of the first spike latency was quantified as the standard deviation of the differences between first spike latencies of the two simultaneously recorded neurons across 10 stimulus repetitions.

**Pooling**: For calculating the stimulus detection accuracy $d_a$ and for neural model simulations, all recordings for a given olfactory receptor neuron type (OR22a or OR59b neurons) were pooled and trials were randomly selected from that pool. Each pool consisted of at least 10 trials with 10 renditions each, resulting in at least 100 spike trains for any given combination of receptor neuron type, odorant and concentration. This pooling procedure is justified, because spikes are independent in olfactory receptor neurons (Kazama and Wilson, 2009).

**Stimulus detection accuracy $d_a$**: Detection accuracies were calculated in a similar way as described by (Jeanne and Wilson, 2015). For each $d_a$ value the following procedure was performed: From the pool of all measurements 40 combinations of n neurons were randomly selected (with n being the number given in the Figures). For each combination, all spikes in the specified integration time windows were summed up in order to obtain spike count distributions over the 40 combinations. For the distribution of odor-evoked spike counts, the window latency and length is given in the respective Figures. For the distribution of spontaneous spike counts all possible non-overlapping windows of given length in the 3 seconds before stimulus trigger were used. Using the mean and standard deviation of those distributions, $d_a$ was calculated as the difference of means divided by the root-mean-square standard deviation:

$$d_a = \frac{\mu_{evoked} - \mu_{spontaneous}}{\sqrt{\frac{1}{2}\left(\sigma^2_{evoked} + \sigma^2_{spontaneous}\right)}}$$

This procedure was repeated 50 times in order to obtain mean and standard deviation of detection accuracy.

We used $d_a > 5$ as threshold for reliable separation of odorant-evoked and spontaneously generated spikes. To estimate the false-positive rate at $d_a > 5$ we calculated 315 spike count distributions for $d_a$ values between 4 and 5 across all recordings. The overlap between spike count distributions of odor-evoked and spontaneous spikes was 0.08 ± 0.36 % (mean ± SD). Thus, at $d_a > 5$, the false-positive rate is less than 1%.

**Spike probability**: The spike probability during a sliding, 1 ms-wide window (rectangular kernel, 0.04 ms step size) was determined over 10 stimulus repetitions. This spike probability was then averaged over all recordings of the same receptor neuron type, odorant and concentration (Figure 1C, Figure S1C).

**Statistical analysis**: Statistical significance of differences in first spike latency (Figure 1D, Figure S2), first spike latency jitter (Figure 1A, Figure S2), spike rate (Figure 1F, Figure S2), integration time to $d_a > 5$ (Figure 2D) and minimum latency to $d_a > 5$ (Figure S4) across different concentrations was assessed using a Wilcoxon signed rank test and a *post-hoc* Holm-Bonferroni correction (* $p<0.05$, ** $p<0.01$, *** $p<0.001$).

**Classification by threshold** (Figure 3C): We used a threshold to predict odorant identity based on the difference in first spike latencies (Figure 3A) or the difference in minimum interspike intervals (Figure 3B) between OR22a and OR59b neuron responses. All possible such differences in the training set (80% of the data) were used to determine a single threshold that classified both odorants across all concentrations best. The threshold classifier was then applied to the test set (the remaining 20% of the data) and the percentage of correct classifications was calculated (classification rate). A classification rate of 50 % corresponds to chance classification. The process was repeated for 100 randomly selected training and test sets.

**Modelling/Simulations**: To classify odorants based on measured receptor neuron responses, a network of 5 spiking neurons, which was inspired by the olfactory system of the fly (Figure 3D, 3E, Figure S5), was simulated using the Python-based simulator Brian2 (integration using Euler method with dt = 25 µs). The network consists of two model Kenyon cells ($KC_X$ and $KC_Y$) that receive weighted input from two model projection neurons ($PN_X$ and $PN_Y$) and one inhibitory neuron (I). The inhibitory neuron is excited by projection neurons and inhibits Kenyon cells (feed-forward inhibition). As input to the projection neurons, recorded spike trains of 23 OR59b and 8 OR22a neurons were selected randomly from the pool of all recordings. Odorant classification was correct when methyl butyrate induced a spike response in $KC_X$ but not in $KC_Y$, and when ethyl acetate induced a spike response in $KC_Y$ but not in $KC_X$. Classification rate was calculated as the percentage of correct classifications during 100 simulation runs for each odorant. All neurons were simulated as leaky integrate-and-fire (LIF) neurons with conductance-based synapses (without synaptic transmission delays) and conductance transients (EPSG) described by an alpha function (Yim et al., 2014). The membrane potential $V_m$ is given by:

$$C_m \frac{dV_m(t)}{dt} = -G_{syn}(t)(V_m(t) - V_{rev}) - G_l(V_m(t) - V_{res})$$

When the threshold was reached, $V_m$ was reset and kept at the resting potential $V_{rest}$ for the duration of the refractory period. The synaptic conductance $G_{syn}$ is the sum of the presynaptic spikes convolved with the alpha function kernel ($t_k$ denotes the spike time of the $k^{th}$ presynaptic neuron):

$$G_{syn} = \sum_k g_{syn}(t - t_k)$$

$$g_{syn}(t) = \begin{cases} J\frac{t}{\tau}e^{1-\frac{t}{\tau}} & if \quad t \geq 0 \\ 0 & if \quad t < 0 \end{cases}$$

The parameters of the neurons were chosen according to the literature (Table S1).

Note, that for the sake of simplicity we omitted several properties of the *Drosophila* olfactory system. For example, 1) we did not include laterality in our model. However, in contrast to most other insects, in *Drosophila*, olfactory receptor neurons project bilaterally to both antennal lobes (Couto et al., 2005), and compared to contralateral receptor neurons, spikes of ipsilateral receptor neurons arrive 0.8 ms earlier at projection neurons, release more transmitter and can evoke 2.5 ms shorter first spike latencies in projection neurons (Gaudry et al., 2013). 2) We restricted the inputs to Kenyon cells to two projection neurons only. However, in *Drosophila*, their number varies from 2 to 12 (Caron et al., 2013; Gruntman and Turner, 2013). 3) Our model Kenyon cells require inputs from 2 projection neurons in order

to generate spikes. However, in *Drosophila* some Kenyon cells can even respond to the input of a single projection neuron (Gruntman and Turner, 2013). 4) We did not implement Kenyon cell driven feedback inhibition onto Kenyon cells themselves (Lei et al., 2013; Lin et al., 2014; Papadopoulou et al., 2011).

## Supplemental References


Caron, S.J.C., Ruta, V., Abbott, L.F., and Axel, R. (2013). Random convergence of olfactory inputs in the *Drosophila* mushroom body. Nature *497*, 113–117.

Couto, A., Alenius, M., and Dickson, B.J. (2005). Molecular, anatomical, and functional organization of the *Drosophila* olfactory system. Curr. Biol. *15*, 1535–1547.

Münch, D., and Galizia, C.G. (2016). DoOR 2.0 - Comprehensive mapping of *Drosophila melanogaster* odorant responses. Sci. Rep. *6*, 21841.

Szyszka, P., Demmler, C., Oemisch, M., Sommer, L., Biergans, S., Birnbach, B., Silbering, A.F.A.F., and Galizia, C.G.G. (2011). Mind the gap: olfactory trace conditioning in honeybees. J. Neurosci. *31*, 7229–7239.

Turner, G.C., Bazhenov, M., and Laurent, G. (2008). Olfactory representations by *Drosophila* mushroom body neurons. J. Neurophysiol. *99*, 734–746.

Yim, M.Y., Kumar, A., Aertsen, A., and Rotter, S. (2014). Impact of correlated inputs to neurons: modeling observations from in vivo intracellular recordings. J. Comput. Neurosci. *37*, 293–304.
